# Preliminary clinical performance of a Cas13a-based lateral flow assay for detecting *Neisseria gonorrhoeae* in urine specimens

**DOI:** 10.1128/msphere.00677-24

**Published:** 2024-12-17

**Authors:** Lao-Tzu Allan-Blitz, Gordon Adams, Gabriela Sanders, Palak Shah, Krithik Ramesh, Jana Jarolimova, Kevin L. Ard, John A. Branda, Jeffrey D. Klausner, Pardis C. Sabeti, Jacob E. Lemieux

**Affiliations:** 1 Division of Global Health Equity, Department of Medicine, Brigham and Women’s Hospital, Boston, Massachusetts, USA; 2 Broad Institute of Massachusetts Institute of Technology and Harvard, Boston, Massachusetts, USA; 3 Division of Infectious Diseases, Department of Medicine, Massachusetts General Hospital, Boston, Massachusetts, USA; 4 Massachusetts Institute of Technology, Boston, Massachusetts, USA; 5 Department of Pathology, Massachusetts General Hospital, Boston, Massachusetts, USA; 6 Department of Population and Public Health Sciences, Keck School of Medicine, University of Southern California, Los Angeles, California, USA; 7 Department of Organismic and Evolutionary Biology, Harvard University, Cambridge, Massachusetts, USA; 8 Department of Immunology and Infectious Disease, Harvard T.H. Chan School of Public Health, Boston, Massachusetts, USA; 9 Howard Hughes Medical Institute, Chevy Chase, Maryland, USA; The University of Iowa, Iowa City, Iowa, USA

**Keywords:** *Neisseria gonorrhoeae*, point of care diagnostic, lateral flow, clinical specimens, CRISPR, Cas13a

## Abstract

Nucleic acid amplification testing (NAAT) for *N. gonorrhoeae* is unavailable in resource-limited settings. We previously developed a CRISPR-based lateral flow assay for detecting *N. gonorrhoeae*. We aimed to pair that assay with point-of-care DNA extraction, assess performance in clinical urine specimens, and optimize assay kinetics. We collected urine specimens among men presenting with urethritis enrolling in a clinical trial at the Massachusetts General Hospital Sexual Health Clinic. We assessed the quantified DNA yield of detergent-based extractions with and without heat. We selected one detergent for extracting all specimens, paired with isothermal recombinase polymerase amplification for 90 minutes and lateral flow Cas13a detection, interpreted via pixel intensity analysis. We also trained a smartphone-based machine-learning model on 1,008 images to classify lateral flow results. We used the model to interpret lateral flow results from the clinical specimens. We also tested a modified amplification chemistry with a second forward primer lacking the T7-promoter to accelerate reaction kinetics. Extraction with 0.02% Triton X resulted in an average DNA yield of 2.6 × 10^6^ copies/µL (SD ± 6.7 × 10^5^). We treated 40 urine specimens (*n* = 12 positive) with 0.02% Triton X, and using quantified pixel intensity analysis, the Cas13a-based assay correctly classified all specimens (100% agreement; 95% CI 91.2%–100%). The machine-learning model correctly classified 45/45 strips in the validation data set and all 40 lateral flow strips from clinical specimens. Including the second forward primer reduced incubation time to 60 minutes. Using point-of-care DNA extraction, our Cas13a-based lateral flow *N. gonorrhoeae* assay demonstrated promising performance among clinical urine specimens.

**IMPORTANCE:**

Using a CRISPR-based assay we previously developed for *Neisseria gonorrhoeae* detection, we developed new techniques to facilitate point-of-care use. We then demonstrated the promising performance of that assay in clinical specimens. Furthermore, we developed a smartphone-based machine learning application for assisting interpretation of lateral flow strip results. Such an assay has the potential to transform the care of sexually transmitted infections in low-resource settings where diagnostic tests are unavailable. A point-of-care pathogen-specific assay, paired with the connectivity offered by a smartphone application, can also support public health surveillance efforts in such areas.

## INTRODUCTION

The prevalence of *Neisseria (N.) gonorrhoeae* infection continues to rise worldwide (1). Resource-limited settings, the areas with the highest burden of infection (2–4), often lack sufficient laboratory infrastructure to diagnose the infection using nucleic acid amplification testing (NAAT) (5, 6). *N. gonorrhoeae* culture, while low-cost, is too time- and labor-intensive to be clinically useful (7). Thus, low-resource settings utilize syndromic management, in which patients with symptoms of urethritis and cervicitis are treated empirically.

Syndromic management misses a high proportion of asymptomatic gonorrhea cases (8–11) and contributes to the emergence of antimicrobial resistance in *N. gonorrhoeae via* the overuse of antibiotics (12, 13). Consequently, low-resource areas may have some of the highest rates of antimicrobial-resistant *N. gonorrhoeae* infection (14–16)—an urgent global public health threat (17, 18). The World Health Organization (WHO) recently declared drug-resistant *N. gonorrhoeae* to be one of seven high-priority organisms (19).

Novel point-of-care diagnostics for *N. gonorrhoeae* are becoming increasingly available (20–22). Some of those tests appear to be both feasible and acceptable in low-resource settings (23). However, few have met WHO standards: real-time connectivity, ease of specimen collection, affordable, sensitive, specific, user-friendly, rapid and robust, equipment-free/environmentally friendly, deliverable to end-users (REASSURED) (24).

We recently reported on the development of a Cas13a-based test for detecting *N. gonorrhoeae* (25). Cas-based assays can be converted into point-of-care tests *via* reagent lyophilization, permitting field deployment (26, 27). Furthermore, our previously reported *N. gonorrhoeae* detection assay demonstrated 100% *in vitro* concordance with reference testing in purified isolates. That assay, however, used DNA extraction techniques which required laboratory training and infrastructure and has not been evaluated in clinical specimens.

This manuscript describes the use of point-of-care DNA extraction techniques paired with Cas13a detection in clinical urine specimens, as well as improvements in assay kinetics to reduce the time to result.

## MATERIALS AND METHODS

### Specimen collection

We enrolled male participants presenting with symptoms of urethritis in the Massachusetts General Hospital Sexual Health Clinic as a part of an ongoing clinical trial (NCT05564299). We enrolled patients between March 2023 and April 2024 if they reported urethral discharge or dysuria, were 18 years of age or older, had no known exposure to *N. gonorrhoeae* or *Chlamydia (C.) trachomatis* within the past 6 weeks, had no concurrent symptoms at extragenital sites, and provided informed consent.

All participants received standard-of-care diagnostics and therapeutics, which included point-of-care Gram stain to evaluate for leukocytes and intracellular diplococci performed on a concurrently collected urethral swab specimen and NAAT using the Aptima Panther System (Hologic, United States). We sent urine specimens for culture with susceptibility testing using standard methods. We considered specimens to be *N. gonorrhoeae* positive if NAAT results were positive.

We aliquoted 1.5 mL of the first-catch urine specimen collected for clinical purposes from male participants’ urine using a sterile pipette into CryoTube Vials (ThermoFisher, United States). We assigned a unique study identifier to each specimen and stored all specimens at −80°C within 30 minutes of collection. For each specimen, we made separate aliquots of working stocks to minimize freeze-thaw cycles and reduce contamination risk. Neither providers nor patients were informed of Cas13a test results.

### DNA extraction

Thermal-based nucleic acid extraction has been recently used for SARS-CoV-2 polymerase chain reaction (PCR) assays (28, 29), while prior work demonstrated Triton X can disrupt cell membranes while simultaneously solubilizing membrane proteins (30). We thus evaluated detergent-based extraction used with or without heat. In the detergent-alone conditions, we added Triton X directly to 50 µL of unspun urine samples at a final concentration of 0.01%, 0.02%, or 0.1%, or Tween-20 at a concentration of 0.02%. In the detergent with heat experiments, we incubated each condition at 95°C for 1 minute or 5 minutes.

We assessed the efficacy of the various DNA extraction methods using the Qbit 4 portable fluorometer (ThermoFisher, United States) double-stranded DNA high-sensitivity assay. We used 10 µL of sample treated with each extraction condition, and adjusted the final reaction volume to 200 µL with the Qbit dsDNA HS buffer, allowing the samples to equilibrate to room temperature for 2 minutes prior to being read in triplicate. We converted the quantified ng/µL to an estimated copies/µL based on the length in base pairs of the *N. gonorrhoeae* genome and the standardized weight of double-stranded DNA. Given the Qbit 4 does not discriminate between the source of DNA, we standardized the results by subtracting the average DNA copies/µL among three urine specimens negative for *N. gonorrhoeae* or *C. trachomatis* treated with each extraction condition. Specimens with negative standardized values were assumed to have no DNA.

For positive controls, we used two cultured and stored *N. gonorrhoeae* isolates, from which we extracted DNA *via* column extraction using the DNeasy Blood and Tissue Kit (Qiagen, Germany) as previously described (25). We also performed column-based extraction on two clinical urine specimens (both spun and unspun). We used separate aliquots for DNA extraction and detection experiments.

### Lateral flow assay

We then selected one detergent-only condition to treat all clinical urine specimens and evaluated lateral flow detection as previously reported (25). We performed one-pot reactions using 45 nM C2c2 *Lwa*Cas13a (GenScript Biotech Corp, United States) resuspended in 1× storage buffer (SB: 50 mM Tris [pH 7.5], 600 mM KCl, 5% glycerol, and 2 mM dithiothreitol) such that the resuspended protein was at 2.25 µM, 1 U/µL murine RNase Inhibitor (New England Biosciences, United States), 10 U/µL T7 RNA polymerase (Lucigen, United States), 1 µM of a poly-uracil biotinylated FAM reporter (Integrated DNA Technologies, United States), 1× SHINE Buffer (SHINE: 20 mM HEPES (4-(2-hydroxyethyl)−1-piperazineethanesulfonic acid) (pH 8.0), 60 mM KCl, and 5% polyethylene glycol), and 2 mM of each ribonucleotide tri-phosphate bases (rNTPs) (New England Biosciences, United States).

We rehydrated the TwistAmp Basic Kit lyophilized recombinase polymerase amplification (RPA) pellets (1 pellet per 73.42 µL master mix volume) using the prepared master mix. We then added 14 mM MgAOc (TwistDx, United Kingdom) after resuspension to activate the RPA pellets. Subsequently, we subdivided the master mix for each guide-primer set pair being analyzed, to which we added 22.5 nM guide RNA (crRNA) and 320 nM each of the RPA primers.

We then incubated the samples at 37°C for 90 minutes, following which we added 80 µL of HybriDetect assay buffer to each sample in a 1:5 dilution along with a HybriDetect lateral flow strip (Milennia Biotec, Germany). We took images using a smartphone camera 3–5 minutes after the strips were added. We performed three tests per specimen in duplicate, and present representative images.

### Lateral flow strip interpretation using pixel quantification

We used quantified pixel intensity to evaluate the lateral flow strips. Using images obtained with an iPhone 12 Pro (Apple Inc., United States), we quantified the pixel intensity with Image J (National Institutes of Health, United States). We imported each photo and standardized it to a 16-bit image. We then calibrated each image and inverted the brightness to report the darker bands as a positive number. Subsequently, we manually determined a bounding box around the test-band regions by visual inspection and calculated the mean pixel intensity. To account for varying brightness due to camera angle and lighting, we subtracted from the average pixel intensity of the test band the average of a distal (non-test) region.

To investigate whether pixel intensity was sufficient for a machine learning-based classification platform, we curated a training data set of 262 lateral flow images. We manually determined a bounding box around each strip and labeled each image with the corresponding class (“positive” or “negative”) using Roboflow (Roboflow, United States). To improve the model’s ability to handle various real-world camera angles, we produced three augmented images per original image, including horizontal flips, rotations (±15°), and shear (±10° horizontally and vertically).

To ensure image quality and optimize our evaluation data set, we developed a binary image classifier to identify and exclude images of poor quality. To do so, we used Image Feature Print V2 (Apple Inc., United States), an existing, pretrained model for preprocessing and extracting salient features of a given image, which we then input into a MobileNet classifier (31). The MobileNet classifier is an existing convolutional neural network model that determines whether images meet predefined quality and framing criteria (images with less than three strips, full visibility of the strip, and predetermined standards for high resolution and focus). We then trained the classifier on a data set of 170 images (85 original and 85 augmented images). We evaluated the performance of the quality classifier model on a validation data set of 100 images (50 of high quality and 50 of low quality).

We then curated an image data set of 1,008 lateral flow strips (*n* = 252 original, *n* = 756 augmented). We trained a model using YOLO v2 (You Only Look Once version 2) architecture (32) on 96% of those images (*n* = 968) without a pretrained vision backbone, preserving *n* = 37 mages for validation. YOLO v2 is a small, fully convolutional neural network designed for real-time object detection that processes an entire image in a single forward pass, making it efficient for real-time applications. We trained the YOLO v2 model using Apple’s CreateML framework (Apple Inc., United States) with epochs of 1,000 (or the number of times the model works through the entire training set) for each batch of images (*n* = 4 images per batch; 1 original and 3 augmented). We elected to use a higher number of epochs given prior work demonstrating improved performance when training models on data sets of <1,000 inputs, particularly among models without pre-training (33). We then applied the model to the validation images and evaluated model performance at 50 epoch intervals to a maximum of 6,000. We selected the model that optimized the mean intersection of union (bounding box accuracy) and classification accuracy (percent agreement with visual classification) and evaluated the final model on a test set of 50 lateral flow strips.

Finally, we implemented the model as a native iOS application *via* Xcode for on-device inference and applied the object detection model to an independent set of lateral flow images from the clinical specimens (two strips per specimen; *n* = 80). We uploaded the code used to GitHub (available at: https://github.com/krithik-ramesh/lateral_flow).

### Augmenting reaction kinetics

To evaluate augmented incubation kinetics, we performed two series of experiments. First, we randomly selected four positive and two negative specimens and repeated the lateral flow detection using the selected detergent with incubation times of 10, 30, 60, and 90 minutes. We quantified the average pixel intensity among the duplicate strips, subtracted the background pixel intensity, as above, and plotted the run kinetics over time. We then repeated the serial incubations using the same four clinical specimens with the addition of a second forward primer lacking the T7-promoter sequence; the second forward primer has been shown to free RPA from competition with T7 transcription by way of generating an additional template for both to proceed (34). We compared average pixel intensity at each timepoint between the two conditions using t-tests in STATA version 17.0 (StataCorp LLC., United States).

### Confirmatory PCR

We used PCR for confirmatory testing of positive clinical specimens (primers and probe sequences shown in the Supplemental Table in the Appendix). To keep PCR analyses consistent with Qbit analyses, we used unpurified urine specimens treated with the selected detergent. We combined TaqPath 1-Step Master Mix (ThermoFisher, United States), 183 nM of each primer, 207 nM of the probe, and DNA template in a 1:2 template to master mix ratio. We adjusted the final reaction volume to 10 µL with nuclease-free water and loaded in duplicated on a 384-well plate, which we ran on a QuantStudio 6 (Applied Biosystems, United States) under the following cycle conditions: incubation at 25°C for 2 minutes, followed by heat activation at 95°C for 2 minutes, followed by 40 cycles of a denaturing step at 95°C for 3 seconds and an annealing step at 60°C for 30 seconds. As we anticipated some degree of PCR inhibition with the addition of a detergent and among unpurified urine specimens, and because we were interested in relative abundance across specimens, we determined the average cycle threshold (CT) value of each specimen, rather than DNA copy number, using the Standard Curve module of the Applied Biosystems Analysis Software.

## RESULTS

### Clinical urine specimens

Overall, we collected 41 clinical urine specimens. Among those, 12 (29.3%) were positive for *N. gonorrhoeae* by NAAT, and 5 (12.5%) were positive for *C. trachomatis*. Of the 41 specimens, 25 (61.0%) had leukocytes on Gram stain from a urethral swab, while 13 (31.7%) had intracellular diplococci (Table 1). Five of the 12 positive specimens were sent for culture, and all returned positive.

**TABLE 1:** Laboratory testing results for clinical urine specimens, including Gram stain, culture, Cas13a lateral flow, and reference testing

Specimen ID	Leukocytes on Gram stain	Diplococci on Gram stain	Culture result	Reference testing result for *C. trachomatis*	Cas13a lateral flow result	Reference testing result for *N. gonorrhoeae*	Concentration of DNA (copies/µL)	Cycle threshold value
RST01	No	No	Not sent					
RST03	Yes	Yes	Not sent		Positive	Positive	2.6 × 10^6^	26.0
RST04	Yes	No	Not sent	Positive				
RST05	.	.	Not sent					
RST07	Yes	Yes	Positive		Positive	Positive	2.6 × 10^6^	28.1
RST09	Yes	No	Not sent					
RST10	Yes	No	Not sent					
RST13	.	.	Negative					
RST14	Yes	Yes	Positive		Positive	Positive	3.3 × 10^6^	28.0
RST17	.	.	Not sent					
RST18	.	.	Negative					
RST20	Yes	No	Not sent	Positive				
RST22	Yes	Yes	Positive		Positive	Positive	2.6 × 10^6^	28.9
RST24	No	No	Not sent					
RST25	Yes	Yes	Not sent		Positive	Positive	2.4 × 10^6^	28.6
RST29	Yes	No	Not sent					
RST32	No	No	Not sent					
RST34	Yes	Yes	Positive		Positive	Positive	1.8 × 10^6^	26.9
RST35	Yes	No	Not sent					
RST36	Yes	Yes	Not sent					
RST37	Yes	Yes	Negative					
RST39	Yes	Yes	Not sent		Positive	Positive	Not detected	32.1
RST40	No	No	Not sent					
RST43	Yes	Yes	Not sent		Positive	Positive	3.9 × 10^6^	29.1
RST44	Yes	No	Not sent					
RST45	Yes	No	Not sent					
RST47	.	.	Not sent		Positive	Positive	3.5 × 10^5^	28.0
RST48	No	No	Not sent					
RST52	Yes	No	Not sent					
RST54	No	No	Not sent					
RST55	No	No	Not sent					
RST61	No	No	Not sent					
RST62^*a*^	Yes	No	Not sent	Positive	Positive	Negative	-	
RST64	Yes	Yes	Not sent		Positive	Positive	2.5 × 10^6^	23.3
RST73	Yes	No	Not sent					
RST75	No	No	Not sent					
RST76	Yes	Yes	Not sent	Positive	Positive	Positive	3.4 × 10^6^	29.4
RST78	No	No	Not sent	Positive				
RST79	No	No	Not sent					
RST80	Yes	No	Not sent					
RST82	Yes	Yes	Positive		Positive	Positive	2.9 × 10^6^	27.2

^*a*^ Excluded, as confirmed to be contaminated on PCR.

### DNA extraction

**Fig 1:**
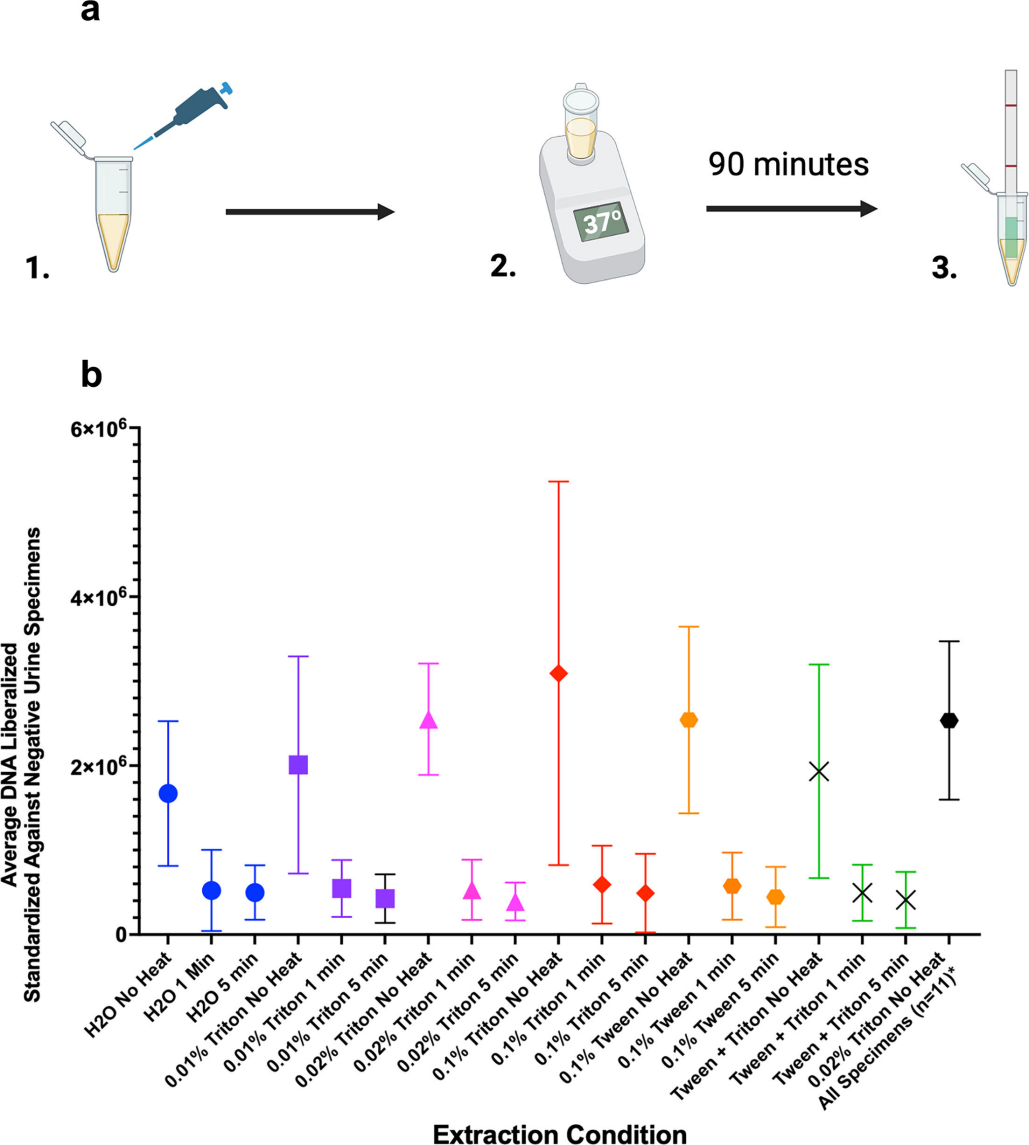
Quantification of *N. gonorrhoeae* DNA extraction comparing detergent-only extraction with combined detergent and thermal extraction. (A) Schematic of the workflow for detergent-based DNA extraction using a clinical urine specimen, followed by incubation at 37°C for 90 min and the Cas13a detection read on lateral flow. (B) Average DNA yield among five positive urine specimens using various extraction conditions standardized by subtracting the average DNA found in three negative urine specimens. The black bar shows the average DNA yield among 11 positive urine specimens, excluding ID039 with undetectable DNA on Qbit fluorometry.

We assessed DNA extraction conditions on a convenience sample of five positive clinical urine specimens and three negative clinical urine specimens (Fig. 1a). Detergent-alone extractions did not differ substantially by DNA yield: 2.0 × 10^6^ copies/µL (SD ± 1.3 × 10^6^; 0.01% Triton X), 2.6 × 10^6^ copies/µL (SD ± 6.7 × 10^5^; 0.02% Triton X), 3.1 × 10^6^ copies/µL (SD ± 2.3 × 10^6^; 0.1% Triton X), 2.5 × 10^6^ copies/µL (SD ± 1.1 × 10^6^; 0.1% Tween), and 1.9 × 10^6^ copies/µL (SD ± 1.3×10^6^; 0.05% Triton X plus 0.05% Tween) (Fig. 1b). The DNA copy number that resulted from combination detergent and thermal conditions was consistently lower than detergent-alone conditions, though not statistically different. We found that column-based extraction among either unspun or spun urine specimens did not yield detectable DNA using the Qbit Fluorometer. Column-based extraction among purified isolates yielded, on average 7.0 × 10^5^ DNA copies/µL (SD ± 2.0 × 10^5^) when extracting a single colony.

As all detergents appeared to perform comparably (Table 1), we selected 0.02% Triton X, which had the narrowest standard deviation, for evaluating extraction among all 12 positive specimens (Table 1; Fig. 1b).

### Lateral flow *N. gonorrhoeae* detection on clinical specimens

**Fig 2:**
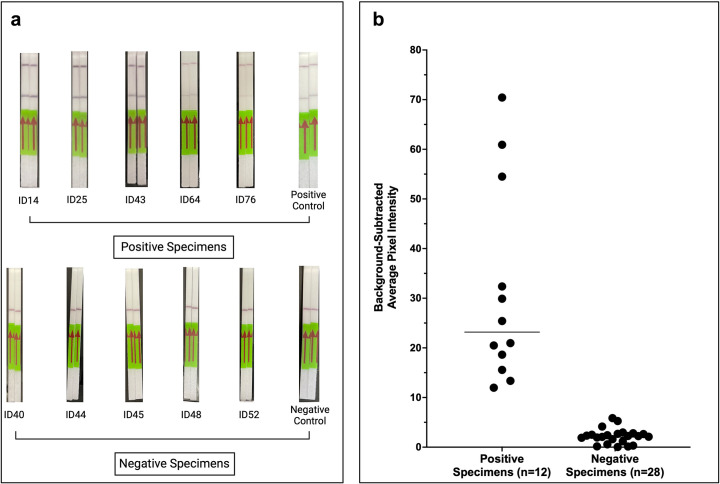
Performance of Cas13a-based lateral flow detection of *N. gonorrhoeae* on clinical urine specimens using detergent-based DNA extraction. (A) Examples of lateral flow results using point-of-care DNA extraction and clinical urine specimens (*n* = 6 positive; *n* = 5 negative; *n* = 1 water control). (B) Mean pixel quantification of images of all lateral flow strips with the background pixel intensity subtracted (*n* = 12 positives; *n* = 28 negatives).

Figure 2a shows an example of lateral flow detection among specimens treated with 0.02% Triton X (Fig. S1 in the Appendix shows images for all specimens). Among the 29 negative specimens, one specimen (ID62) tested negative by NAAT but positive by lateral flow. Confirmatory PCR of specimen ID62 was also positive among three separate aliquots (average CT value 21.2); thus, it was assumed to be contaminated and excluded from the analysis. By visual inspection alone, lateral flow correctly identified 28/28 negative specimens.

Among the 12 positive specimens, two (ID39 and ID47) which tested positive by NAAT, showed faint positive signals on lateral flow; however, on quantitative pixel analysis, the standardized mean pixel intensity for all specimens was above the mean intensity for the negative specimens (Fig. 2b). Specimen ID39 that had no detectable DNA extracted as determined by Qbit and had the highest average CT value (32.1) of all specimens on PCR. Specimen ID47 had 3.5 × 10^5^ DNA copies/µL extracted (average CT value 28.0) (Table 1).

Thus, by quantitative pixel intensity analysis, the Cas13a lateral flow assay correctly identified 12/12 (100%) positive specimens and 28/28 (100%) negative specimens. The overall percent agreement of the Cas13a-based lateral flow assay with reference NAAT was 100% (95% CI 91.2%–100%) (40/40).

Furthermore, the machine-learning model image classifier model (high or low quality) achieved 100% classification accuracy on the validation data set (*n* = 100 images; 50 high quality and 50 low quality). For lateral flow classification, we selected the model at 4,000 epochs, which optimized the mean intersection of union scores and accuracy. Figure S2 shows the learning curve (loss by number of iterations) in the training and validation data sets, which demonstrated consistent decreases in learning, indicating the absence of overfitting. When utilized in iOS application format, the model demonstrated inference speeds of 9 ms on iPhone X and newer models.

Of the 37 images in the validation data set, none were excluded by the quality classifier, and the model correctly classified 19/19 positive strips and 18/18 negative strips (overall agreement 100%; 95% CI 90.6%–100%). Among the final validation data set, five strips were removed due to low resolution, and the model correctly classified 18/18 positive strips and 27/27 negative strips (overall agreement 100%; 95% CI 92.1%–100%).

When evaluating images of lateral flow strips for the 40 clinical specimens (*n* = 80 images as each strip was run in duplicate), the model correctly classified all 80 images as either positive or negative (Fig. S3 shows examples of the iOS interface interpreting positive and negative lateral flow strips).

### Accelerating reaction kinetics

**Fig 3:**
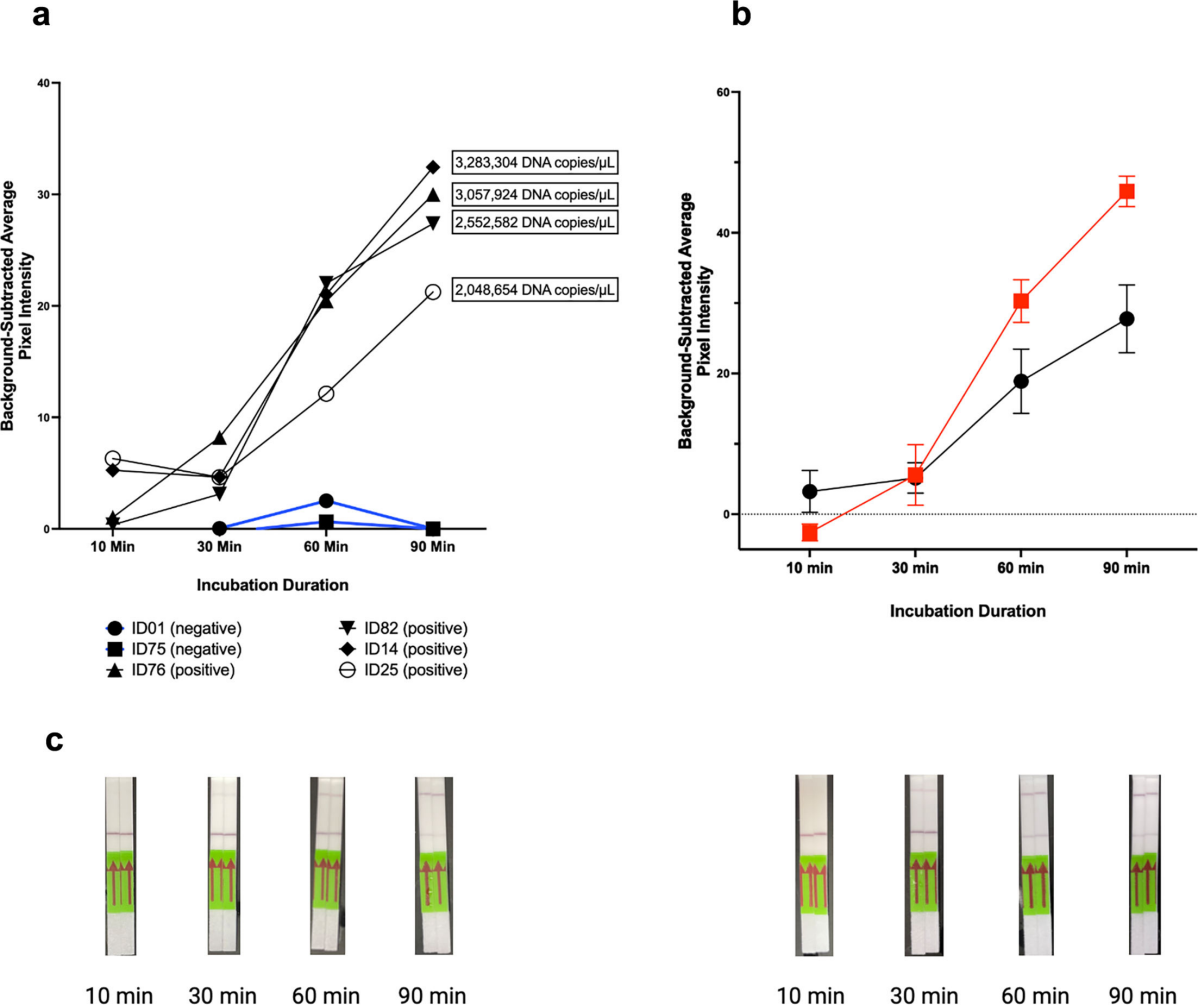
Serial incubation of Cas13a lateral flow *N. gonorrhoeae* detection under standard conditions as well as with additional forward primer lacking a T7 promoter, analyzed using quantitative pixel intensity. (A) Background-subtracted pixel intensity of lateral flow results for positive (*n* = 4) and negative (*n* = 2) clinical urine specimens as measured after 10, 30, 60, and 90 min of incubation. (B) Background-subtracted pixel intensity averaged for four lateral flow results under standard conditions (black circles) and after the addition of a forward primer lacking a T7 promoter (red squares), following the same serial incubation. The average pixel intensity at both 60 min (*P* = 0.01) and 90 min (*P* = 0.001) was significantly higher when a forward primer lacking the T7 promoter was added. (C) Examples of the lateral flow strips for both standard conditions and after inclusion of the additional forward primer.

Under standard assay conditions, the detection was minimal after 10 and 30 minutes of incubation; while positive bands became visible after 60 minutes, band intensity was greater after 90 minutes (Fig. 3a). After the addition of a second forward primer lacking the T7-promoter, the average pixel intensity was significantly higher after 60 minutes (mean difference 11.4, *P* = 0.01) and 90 minutes (mean difference 18.1, *P* = 0.001) of incubation compared to standard conditions (Fig. 3b). There was no significant difference in mean pixel intensity at 60 minutes among the reactions with the additional forward primer compared to 90 minutes under standard conditions (mean difference 2.5, *P* = 0.41). Thus, the modified assay reduced detection time by approximately 30% (or 30 minutes).

## DISCUSSION

We report on the preliminary performance of a Cas13a-based lateral flow *N. gonorrhoeae* detection assay on clinical urine specimens using point-of-care DNA extraction with a single detergent. Our assay showed 100% agreement with NAAT results when image analysis of the lateral flow strips was used. Point-of-care DNA extraction combined with lyophilization of reagents previously done for Cas-based assays (26, 27) can produce a field-deployable point-of-care molecular test for *N. gonorrhoeae*. Furthermore, we developed a smartphone-based machine-learning model to facilitate test interpretation.

Detergent-based extraction without heat may be more easily deployed in low-resource settings where access to a heat source is limited. Our results indicate that extraction using detergents alone resulted in DNA quantities above the previously determined analytic sensitivity of the assay (25). We were not able to directly compare to column-based extraction given the inherent differences in extracting purified isolates and clinical urine specimens. In addition, the specific detergent, and concentration of the detergent, did not seem to significantly impact the DNA yield among a small convenience sample. Further research may be able to optimize detergent-based extraction.

However, more important than the DNA concentration extracted was whether the quantity of DNA extracted was sufficient for Cas13a detection. We demonstrated that detergent-alone extraction was sufficient for pathogen detection on the samples tested using Cas13a with RPA. While our assay was able to detect specimens with relatively high CT values on PCR (e.g., 32.1), more dilute specimens may pose a challenge for interpretation if the positive bands become faint.

We also demonstrated that reaction kinetics could be augmented via the addition of a forward primer lacking the T7-promoter, decreasing incubation times from 90 minutes to 60 minutes, which is consistent with prior work (34). To meet the standards of the WHO REASSURED criteria (24), more rapid amplification is still be required; other isothermal methods may facilitate further reductions in amplification time, such as loop-mediated amplification (35), helicase-dependent amplification (36), and strand-displacement amplification (37). Optimized DNA extraction methods may also equate to more rapid amplification by making more DNA available.

Our machine-learning model may further support classification in such instances; among the currently available strips, the model demonstrated excellent performance, which is consistent with prior work using similar models for image segmentation and pixel quantification (38–40). In addition, such models when used on smartphone devices, have the potential to not only improve consistency of strip interpretation but also facilitate real-time connectivity, an important component of the WHO REASSURED criteria (24). Further evaluation of the assay configuration paired with the machine-learning model for interpretation in real-world settings is warranted.

### Limitations and future directions

Our results have several limitations. First, we tested our assay on a small number of urine specimens among only men. In addition, the urine samples were frozen prior to analysis, which might affect assay performance. Given the promising performance of the assay in this single-center study, evaluation among larger and more diverse samples is warranted. This was a single pathogen detection system. Simultaneous detection of multiple pathogens (e.g., *C. trachomatis*) will be needed to supplant syndromic management. In addition, the incorporation of molecular targets predicting antimicrobial resistance has the potential to facilitate resistance-guided therapy. Given the potential impact of this assay, we feel the current platform is a promising step toward improving care of *N. gonorrhoeae* infection globally.

### Conclusion

Using point-of-care DNA extraction, isothermal amplification, and Cas13a-based detection, the lateral flow *N. gonorrhoeae* assay reported here demonstrated promising performance on clinical urine specimens. Interpretation of lateral flow results can be supported by machine-learning classification models. Further evaluation of the assay among larger and more diverse samples is warranted.

## SUPPLEMENTAL MATERIAL

Supplemental material msphere.00677-24-s0001.docx Supplemental table and figures.

ASM does not own the copyrights to Supplemental Material that may be linked to, or accessed through, an article. The authors have granted ASM a non-exclusive, world-wide license to publish the Supplemental Material files. Please contact the corresponding author directly for reuse.
