# Supplementary material for: Preliminary clinical performance of a Cas13a-based lateral flow assay for detecting Neisseria gonorrhoeae in urine specimens
Source: mSphere. 2024 Dec 17;10(1):e00677-24. doi: 10.1128/msphere.00677-24 (PMC11774021; doi:10.1128/msphere.00677-24)
Supplement: Supplemental material — Supplemental table and figures. [file msphere.00677-24-s0001.docx]

**Appendix**

**Supplemental Table: Primer and Probe Sequences for Quantitative Polymerase Chain Reaction Used for Detecting the *por*A Gene in *N. gonorrhoeae***

|  | **Sequence** |
| --- | --- |
| Forward Primer | 5’ - TTTTCCGGTTTCAGCGGCAGCATTCAA - 3’ |
| Reverse Primer | 5’ - ATTACTTTCCAGCGTGAAAGTAGCAGGCGT - 3’ |
| Probe | 5’ - ACGCGAACATACCAGCTATGATCAAATC - 3’ |

**Supplemental Figure 1: Lateral Flow Cas13a Detection of *N. gonorrhoeae* Among 40 Clinics Urine Specimens**

**
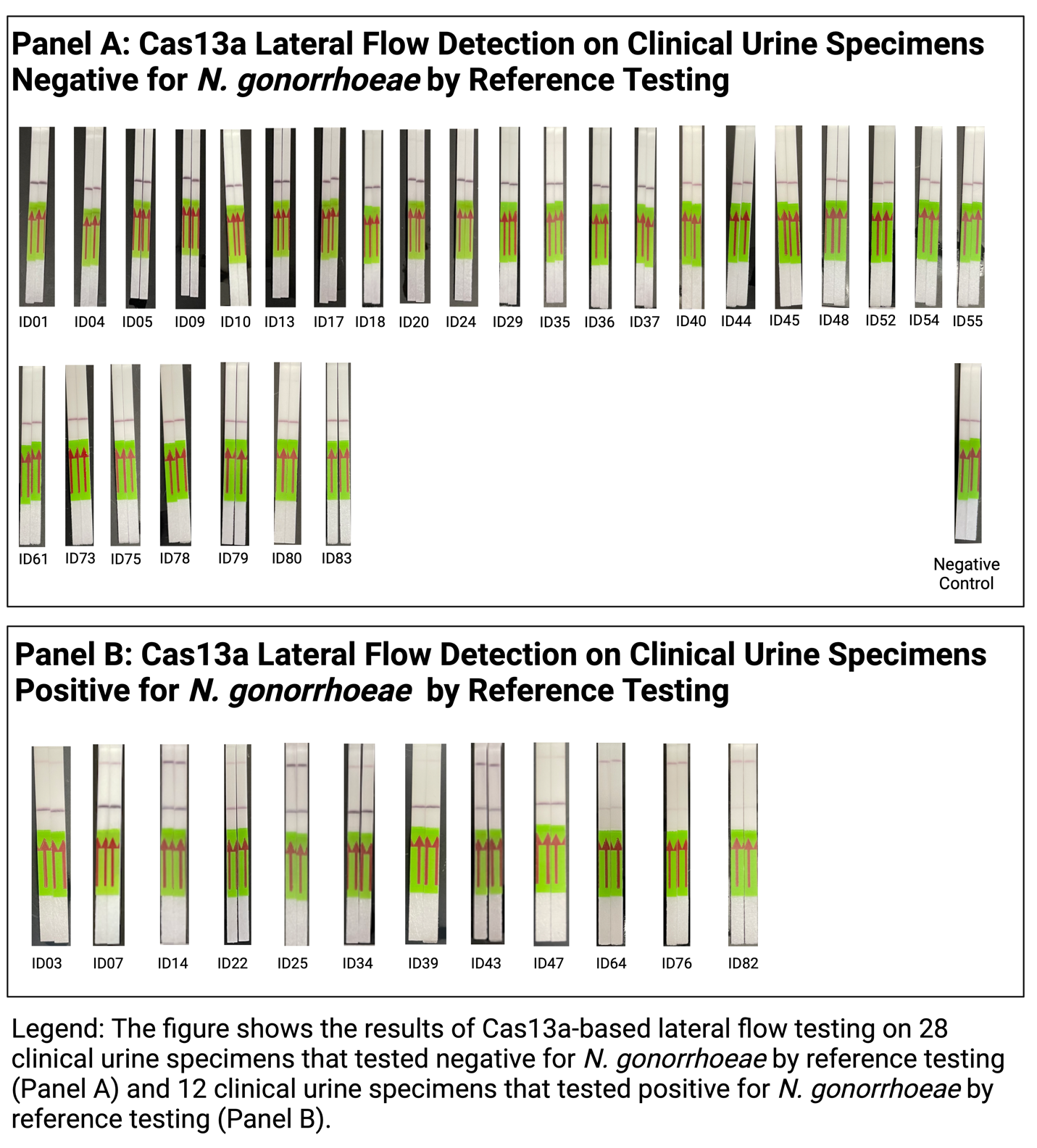
**

**Supplemental Figure 2: Learning Curve Showing Loss in Training and Validation Datasets**

**
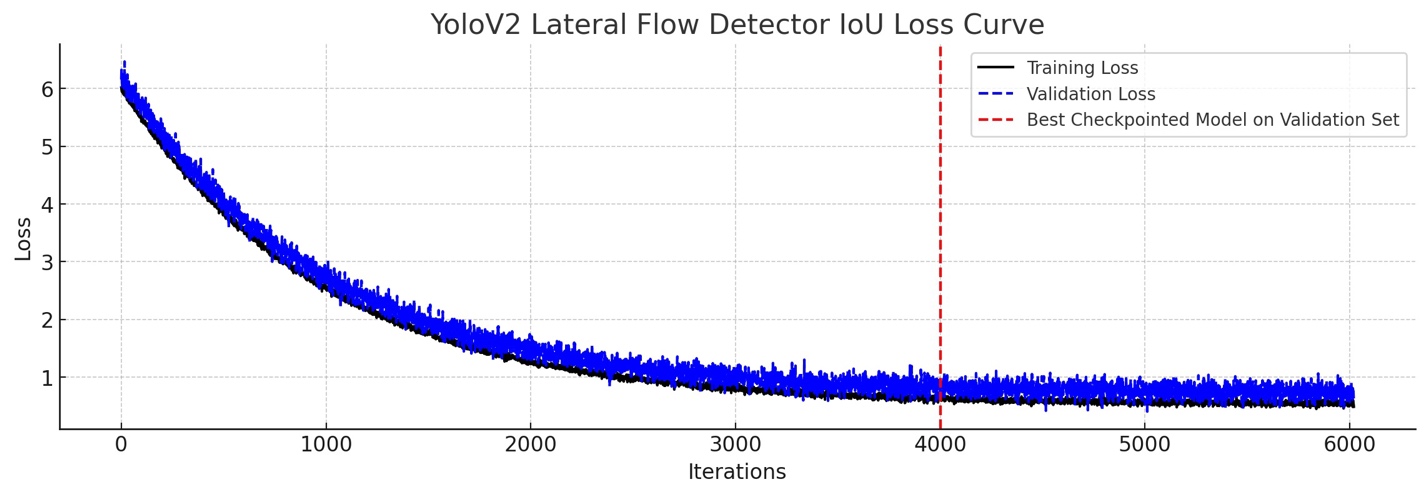
**

Legend: The figure shows the change in loss by epochs for the training dataset (black) and the validation dataset (blue).

**Supplemental Figure 3: Examples of the iOS Interface of the Machine-Learning Application Interpreting Positive and Negative Lateral Flow Strips**

**
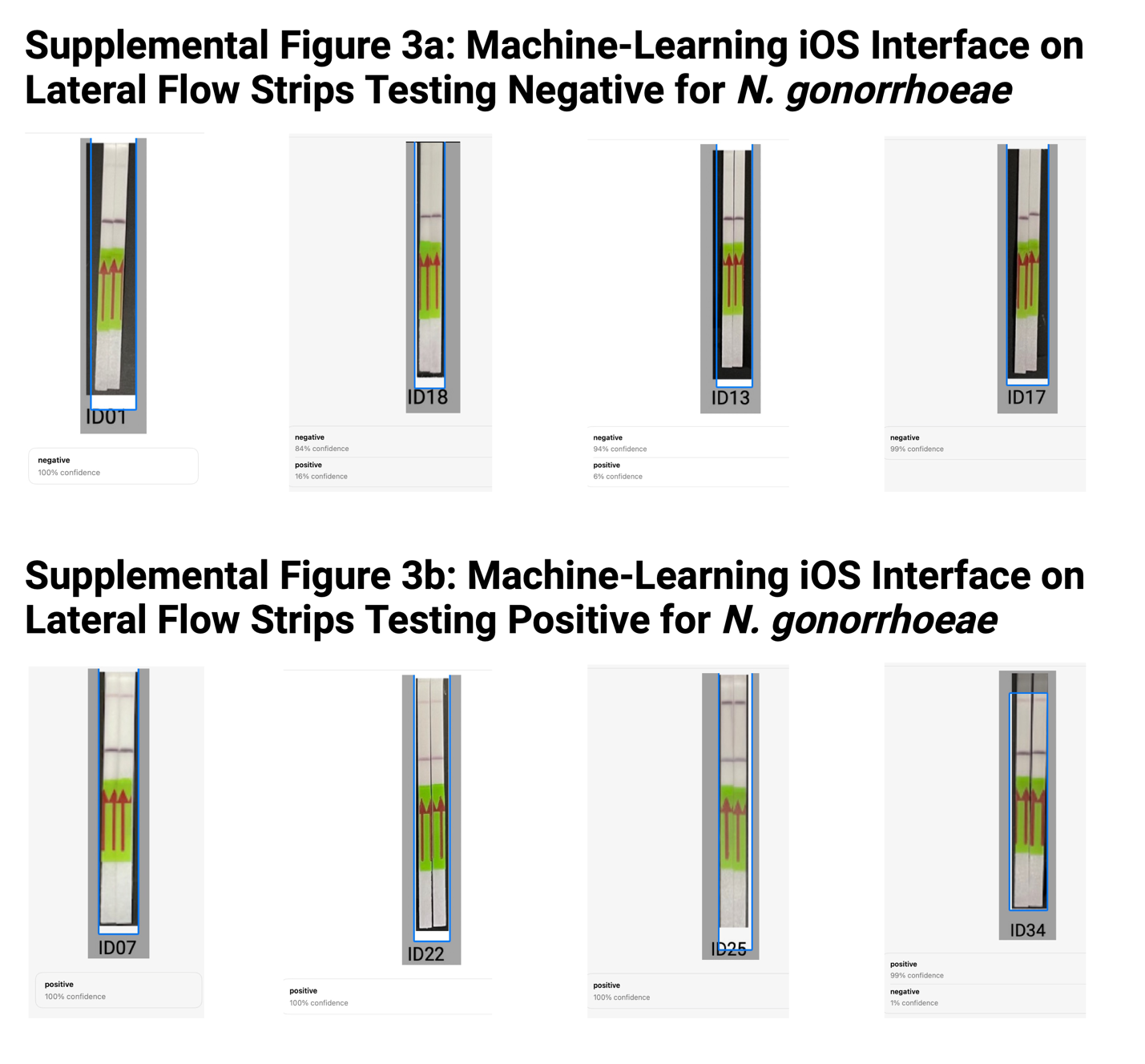
**

Legend: The figure shows examples of the iOS interface of the machine-learning model interpreting lateral flow strips classified as negative (panel A) or positive (panel B).
